# Pharmacokinetic Comparison of Eight Major Compounds of *Lonicerae japonicae flos* after Oral Administration in Normal Rats and Rats with Liver Injury

**DOI:** 10.3390/molecules27238211

**Published:** 2022-11-25

**Authors:** Xuejiao Wang, Songtao Liu, Lin Yang, Jiaojiao Dong, Shihao Zhang, Jiahao Lv, Liu Yang, Hai Jiang

**Affiliations:** 1 Key Laboratory of Basic and Application Research of Beiyao, Heilongjiang University of Chinese Medicine, Ministry of Education, Harbin 150040, China; 2 School of Pharmacy, Jiangxi University of Chinese Medicine, Ministry of Education, Nanchang 330004, China

**Keywords:** *Lonicerae japonicae flos*, liver injury, pharmacokinetics, UPLC-MS/MS

## Abstract

Traditional Chinese medicine considers *Lonicerae japonicae flos* to have antibacterial detoxification, liver protection, and gallbladder protection. At present, studies have proven that *Lonicerae japonicae flos* has a good therapeutic effect on liver injury. Therefore, to confirm the clinical applicability of *Lonicerae japonicae flos* in the treatment of liver injury, we were the first to compare the pharmacokinetics of an oral ethanol extract of *Lonicerae japonicae flos* in normal rats and carbon tetrachloride-induced liver injury model rats. A method was developed for the simultaneous determination of 3-caffeoylquinic acid, 4-caffeoylquinic acid, 5-caffeoylquinic acid, 3,5-dicaffeoylquinic acid, 4,5-dicaffeoylquinic acid, protocatechuic acid, Sweroside, and Secoxyloganin in rat plasma by ultra-performance liquid chromatography tandem mass spectrometry. The results show that the method is reliable and reproducible and can be used for quantitative determination of biological samples. The pharmacokinetic parameters showed that the area under the concentration–time curve of eight compounds in the model group was significantly increased. The results showed that the total absorption of the active components of *Lonicerae japonicae flos* in the blood increased, the clearance rate slowed down, and the bioavailability of *Lonicerae japonicae flos* increased in liver injury diseases.

## 1. Introduction

*Lonicerae japonicae flos* (LJF) is the dried flower bud or the flower with the first opening of Lonicera japonica Thunb [1]. As a national plant medicine, it is widely distributed in Asian countries and has great medicinal value (www.theplantlist.org (accessed on 24 May 2022)). In China, LJF is known as an ancient medicine used to treat fever and in detoxification [2] due to its ventilation and heat dissipation abilities [3]. Modern research has shown that LJF contains a large number of flavonoids [4], iridoids [5], triterpenoid saponins [6], organic acids [7,8], and other compounds [9]. Based on the effective material compounds, modern pharmacology has confirmed that LJF has anti-inflammatory [10], antibacterial [11], antiviral [12,13], and antioxidation [14] properties, as well as it increases immune function [15] and protects the liver and gallbladder [16,17]. Clinically, LJF was a commonly used antipyretic, often used to treat acute upper respiratory tract infections, rashes, a selection of wounds, tonsillitis in children, and other diseases [18,19,20], but the clinical preparation of LJF in protecting the liver and gallbladder has not been found.

The liver is one of the largest organs in the human body, and it is also the main place for metabolism and excretion. It plays a vital role in maintaining bodily functions and regulating the balance of the human body. Current research shows that there are about 2 million new cases of liver disease worldwide each year, and millions of deaths from liver disease related to chemical liver injury, accounting for about 3.5% of all deaths [21]. If liver damage is not promptly treated, it can lead to many forms of liver disease, such as acute and chronic hepatitis, granulomatous hepatitis, cholestasis due to bile duct damage, cholestasis with or without hepatitis, steatohepatitis, vascular disease, and tumors [22,23]. However, at present, chemical drugs are mostly used in the clinical treatment of liver injury, which have obvious side effects and are relatively expensive [24,25]. The curative effect of natural medicine on liver injury-related diseases is better, with less side effects, a range of sources, and a low price, and the experimental and clinical research results show the broad application prospects [26].

At present, the research on the liver and gallbladder protection properties of LJF is mostly pharmacological research. Hu et al. believed that the total flavonoids of LJF had a significant effect on the treatment of immunological liver injury in mice, which could effectively reduce the liver and spleen indexes of mice, and effectively improve the pathological conditions [27]. Chen et al. believed that the extract of LJF could significantly reduce the levels of alanine aminotransferase (ALT) and aspartate aminotransferase (AST) in serum, showing a certain hepatoprotective effect [28]. Wang et al. believe that LJF can inhibit the occurrence of lipid peroxidation and reduce the liver index [29]. The study of pharmacokinetics is helpful to explain the material basis of the actual treatment which is closely related to liver protection. Therefore, studying the pharmacokinetics of LJF against liver injury is of practical significance for the clinical development of new drugs to protect the liver.

So far, most of the studies on the pharmacokinetics of silver anthers have focused on the pharmacokinetics of 3-caffeoylquinic acid (3-CQA) in rats after oral administration [30], but there is no pharmacokinetic study of LJF in the pathological model of liver injury. In this study, the ultra-performance liquid chromatography tandem mass spectrometry (UPLC-MS/MS) method was used to study the pharmacokinetics of 3-CQA, 4-caffeoylquinic acid (4-CQA), 5-caffeoylquinic acid (5-CQA), 3,5-dicaffeoylquinic acid (3,5-diCQA), 4,5-dicaffeoylquinic acid (4,5-diCQA), protocatechuic acid (PA), Sweroside, and Secoxyloganin in the rats with acute liver injury induced by CCl_4_ [31,32,33]. The purpose of this study was to explain and compare the absorption and metabolism of the active ingredients of LJF in normal rats and liver injury model rats, and to provide a theoretical basis for the clinical development and application of new preparations for the liver protection properties of LJF.

## 2. Results

### 2.1. Optimization of Chromatographic Conditions

**Figure 1:**
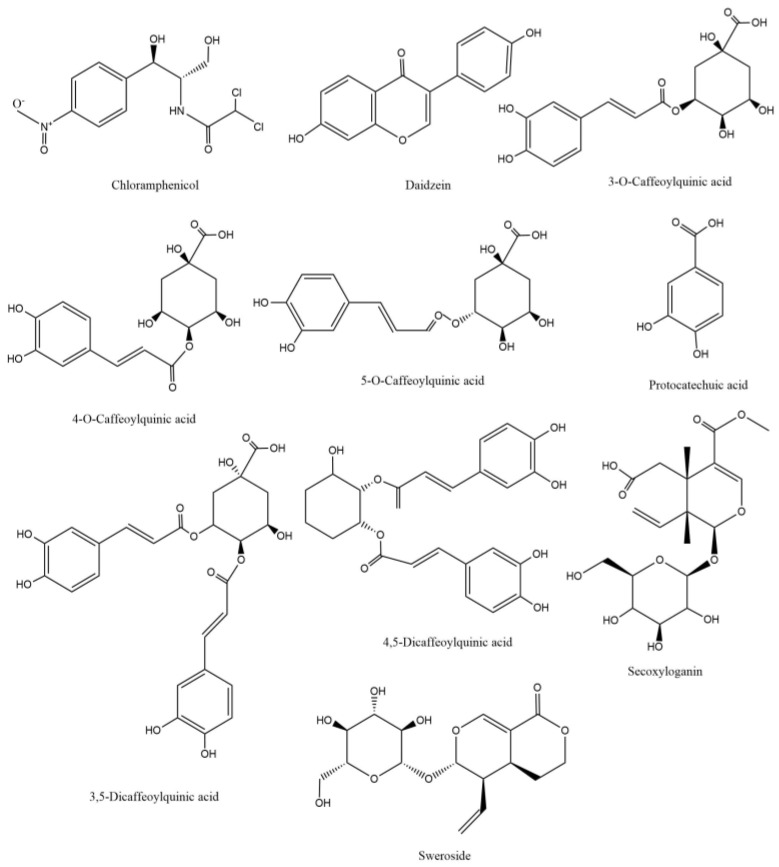
Chemical structures of the eight compounds and two internal standards (ISs).

The selection and optimization of mass spectrum parameters is very important for the quantification of analytes. The results showed that the response values of 3-CQA, 4-CQA, 5-CQA, 3,5-diCQA, 4,5-diCQA, PA, and Secoxyloganin (the structures are shown in Figure 1) under negative ion mode were higher than those under positive ion mode, and the response values of Sweroside under positive ion mode were better (MS/MS detection parameters for eight compounds in Table 1). To improve the chromatographic peak shape, enhance the sensitivity, and shorten the running time, the liquid phase conditions were optimized. Several mobile phase systems were studied, and the best reactivity, sensitivity, and separation efficiency were selected. Methanol–water and acetonitrile–water mobile phase systems with 0.1% formic acid were compared. The results showed that the methanol−0.1% formic acid system had the best chromatographic performance.

### 2.2. Optimization of Sample Preparation

Plasma samples were treated with methanol/acetonitrile and ethyl acetate. The results showed that the recovery of protein precipitated with methanol was higher than that with other solvents, the matrix effect was lower, and the reproducibility was good.

### 2.3. Results of Rat Model of Liver Injury

**Figure 2:**
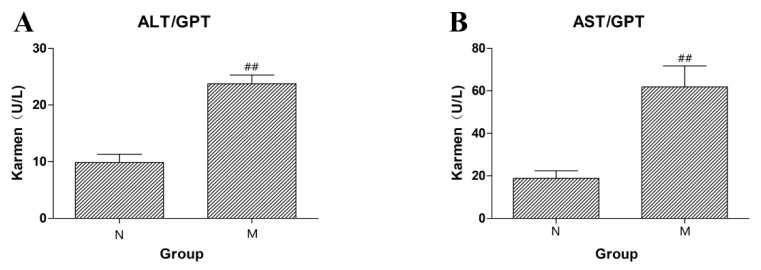
(**A**) The content of ALT in rat serum of the normal control (N) group and the liver injury model (M) group. (**B**) The content of AST in rat serum of the normal control (N) group and the liver injury model (M) group (^##^ *p* < 0.01 compared to N).

Intraperitoneal injection of CCl_4_ was used to induce liver injury in rats, and the successful establishment of the model was measured by detecting ALT and AST in rat plasma. ALT and AST in the model group were significantly increased (as shown in Figure 2, *p* < 0.01). The results showed that the model of acute liver injury was successfully established. 

### 2.4. Methodological Verification

#### 2.4.1. Specificity

**Figure 3:**
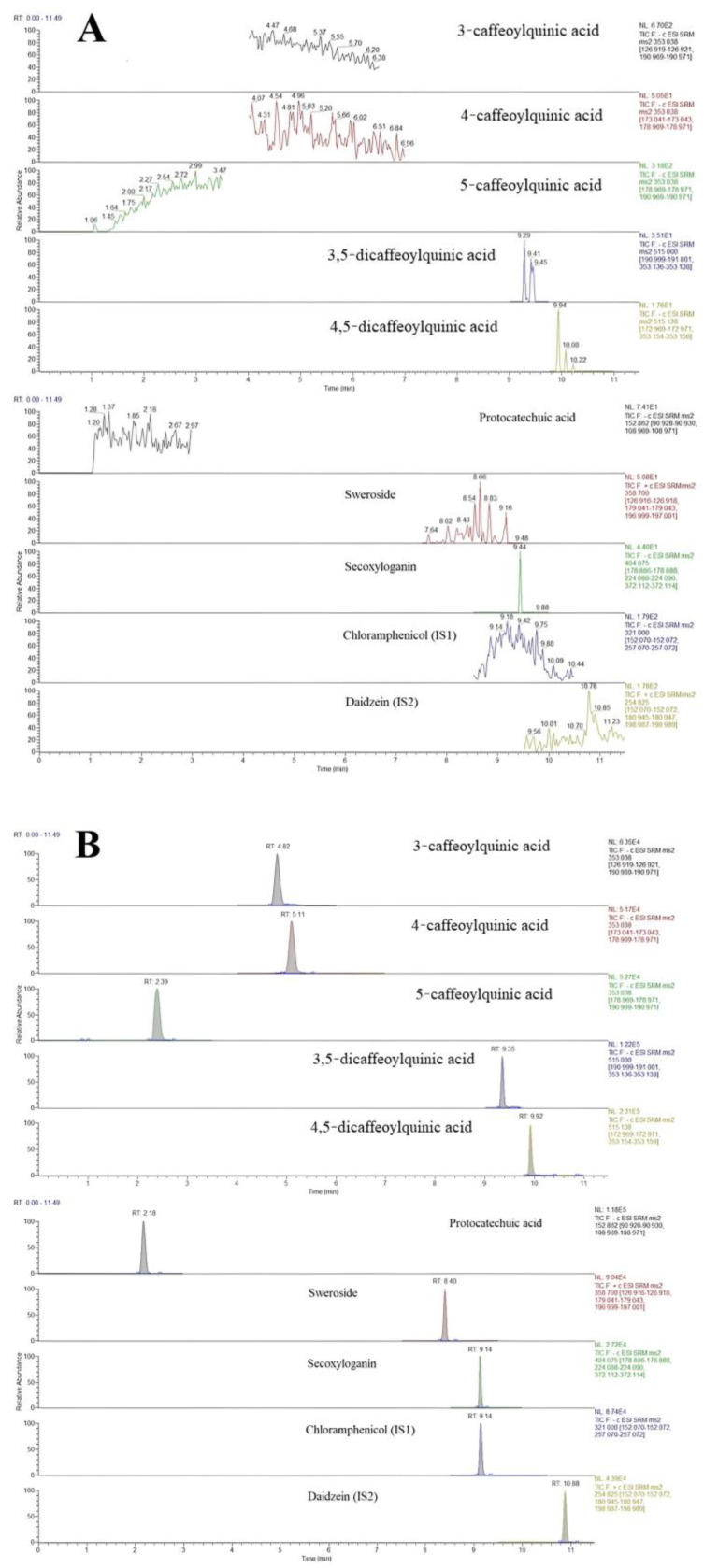

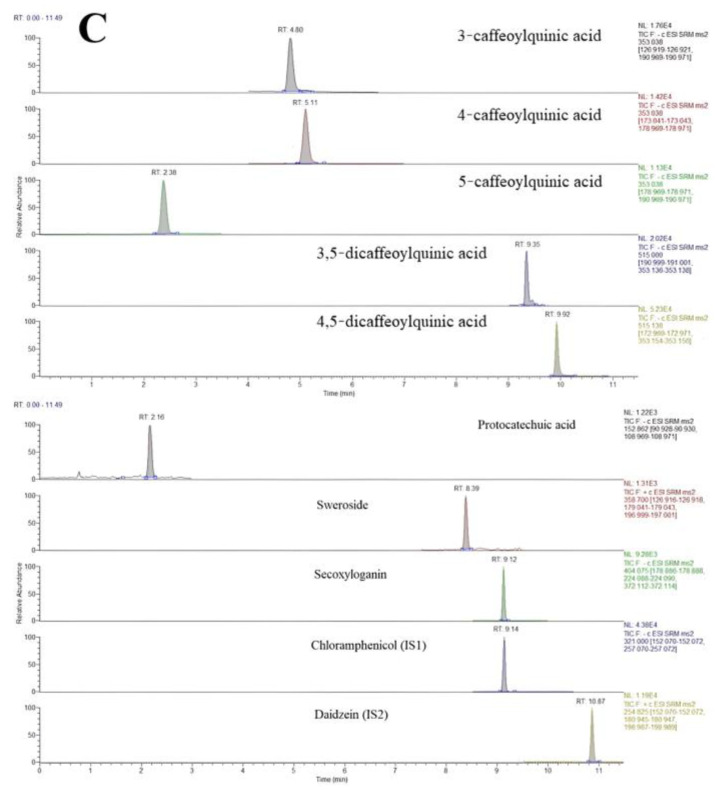
Typical MRM chromatograms of eight analytes and the ISs: (**A**) blank plasma, (**B**) blank plasma spiked with the analytes and IS, and (**C**) plasma samples 5 min after oral administration of the LJF aqueous extract.

The chromatograms of blank plasma samples, blank plasma samples with 8 added analytes and IS1 and IS2, and plasma samples taken orally for 5 min with ethanol extract of LJF are shown in Figure 3. The results showed that the eight active components and the internal standards were not disturbed by endogenous substances in plasma under the corresponding retention time. The method established in this study had good specificity.

#### 2.4.2. Linearity and Lower LLOQ

The eight analytes showed a good linear relationship in rat plasma (r > 0.990). The linear regression equation, linear range, linear regression coefficient, and LLOQ of the active components are shown in Table 2.

#### 2.4.3. Accuracy and Precision

In this experiment, six duplicate QC samples of the eight analytes at three concentration levels (LQC, MQC, HQC) were tested on the same day and on three different days. The intra- and inter-day accuracy and precision of the 8 analytes to be analyzed were less than 15%, respectively, indicating that the established method has good precision and accuracy. The results are shown in Table 3.

#### 2.4.4. Recovery and Matrix Effect

As shown in Table 4, the eight active ingredients and internal standard compounds had good extraction recovery, and plasma had no significant matrix effect on the eight active ingredients and internal standard compounds, which further verified the accuracy and reliability of the established method.

#### 2.4.5. Stability

As shown in Table 5, the eight active ingredients had good stability after being placed at room temperature (25 °C) for 4 h, at −80 °C for 30 days, at −80 °C/room temperature for 3 freeze/thaw cycles, and at 4 °C for 24 h in the sample chamber.

### 2.5. Application in a Pharmacokinetic Study in Rats

**Figure 4:**
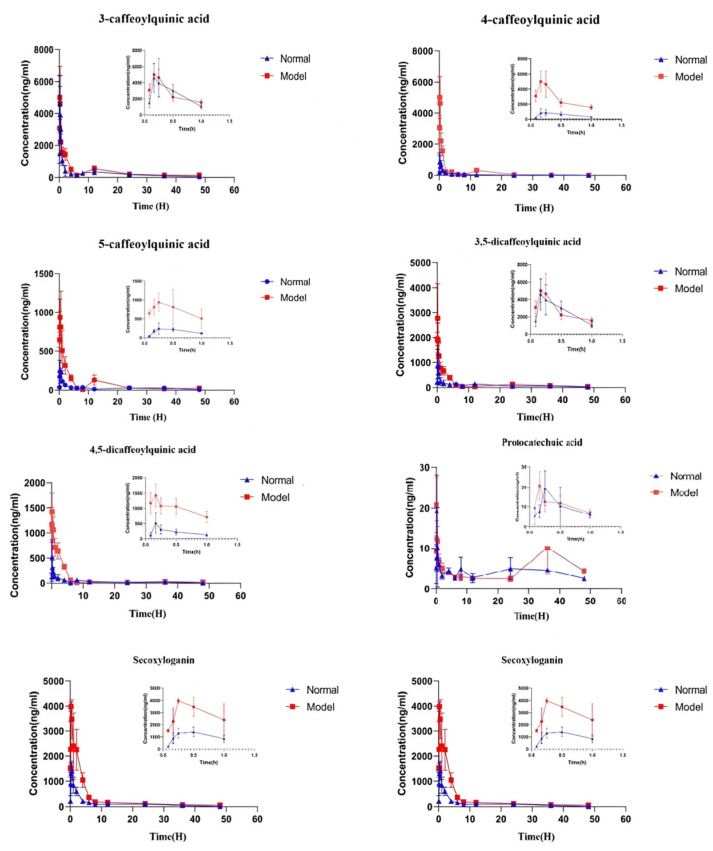
Mean concentration–time curves of the eight compounds in N (normal rats group) and M (liver injury model rat group) rat plasma after oral administration of the LJF aqueous extract. Values are presented as mean ± SD of 6 rats.

This method has been successfully applied to study the pharmacokinetics of LJF orally in normal rats and acute liver injury rats. The pharmacokinetic parameters were analyzed using the non-atrioventricular model analysis on the DAS 2.0 platform. The mean plasma concentration–time curves of 3-CQA, 4-CQA, 5-CQA, 3,5-diCQA, 4,5-diCQA, PA, Secoxyloganin, and Sweroside are shown in Figure 4. Pharmacokinetic parameters included: *C_max_*, *T_max_*, area under the concentration–time curve (*AUC_0–t_*), *AUC_0–∞_*, *MRT_0–t_*, and *MRT_0–∞_*, as shown in Table 6 and Figure 4.

#### 2.5.1. The Pharmacokinetic Characteristics of Phenolic Acids

The pharmacokinetic characteristics of phenolic acids of LGF in plasma of the rat liver injury model were studied for the first time. Compared with the normal group, after oral administration of *Lonicerae japonicae flos*, *C_max_* absorption of 4-CQA and 5-CQA in plasma of rats in the model group increased, and *AUC_0–t_* and *AUC_0–∞_* also increased (*p* < 0.01). *T_max_* of 5-CQA was not significantly different from that of the normal group and *t_1_*_/*2*_ was prolonged, but there was no significant difference. Compared with the normal group, the *T_max_* of 4-CQA was significantly decreased and the *T_max_* was prolonged, but there was no significant difference. *AUC_0–t_* and *AUC_0–∞_* of 3-CQA were significantly increased compared with the normal group (*p* < 0.01), and the *C_max_* value was increased but there was no significant difference. There was no significant difference between *T_max_* and *t_1/2_* compared with the normal group. The *C_max_*, *AUC_0–t_*, and *AUC_0–∞_* of 3,5-diCQA and 4,5-diCQA increased significantly compared with the normal group. The *t_1/2_* of 4,5-diCQA was prolonged, with a significant difference, and the value of *MRT_0–∞_* was significantly increased compared with the normal group. The *AUC_0–t_* of 3,5-diCQA was significantly increased compared with the normal group (*p* < 0.05). Compared with the normal group, the *T_max_, AUC_0–∞_*, and *MRT_0–t_* of the PA model group reached the peak earlier, the area under the curve of the drug duration increased, the retention time was prolonged, and the clearance rate decreased.

The results showed that under the condition of liver injury, the value of *AUC_0–∞_* of phenolic acids in LJF increased (*p* < 0.05), and the amount of exposure in vivo was significantly increased compared with the normal group.

#### 2.5.2. Pharmacokinetic Characteristics of Iridoid Glycosides

The *AUC_0–∞_* value of Sweroside in the model group was significantly higher than that in the normal group (*p* < 0.01) and the *C_max_* value was increased compared with that in the normal group, but there was no significant difference. *C_max_* and *MRT_0–∞_* of Secoxyloganin were significantly higher than those in the normal group (*p* < 0.01), but *T_max_* was significantly lower than that in the normal group (*p* < 0.05).

The results showed that compared with the normal group, the clearance rate of iridoid glycosides in the model group was lower and the absorption amount was increased, which may be beneficial for the treatment of liver injury.

## 3. Discussion

Based on the hepatoprotective effect of LJF, it is of great significance to investigate the pharmacokinetics of LJF in vivo in normal and liver injury model groups. In this study, the UPLC-MS/MS method was established to study the pharmacokinetic parameters in normal rats and rats with liver injury. The experimental results showed that the pharmacokinetic parameters in normal rats and model rats were significantly different after a single administration of LJF extract (*p* < 0.01, *p* < 0.05). Combined with the current research status, the reasons may be as follows.

First, it may be related to the effect of liver injury on CYP450 enzyme activity and abnormal biliary excretion in vivo [34,35,36,37]. The liver CYP450 system metabolic enzyme is the main pathway of drug metabolism. Liver injury caused by CCl_4_ will affect the liver CYP450 system metabolic enzyme, resulting in decreased activity of drug metabolism enzymes, which leads to increased drug absorption, a slow elimination rate in the body, and increased total drug absorption into the blood. In addition, bile acids (BAS) are closely related to the metabolic function of the liver. In the pathological state of the liver, BAS metabolism disorder is manifested as an increased BAS level in patients’ serum and an abnormal composition proportion of BAS metabolites, which can feedback-regulate the enterohepatic circulation of BAS and the progress of liver disease by affecting the functions of immune cells and liver cells, thus affecting the activity of drug metabolism enzymes.

Secondly, based on the enteric–hepatic axis theory, the drug absorption and metabolism under the liver injury disease model were explained to provide new ideas and strategies for the treatment of liver injury [38]. On the one hand, the gut-liver axes directly affects through the entero-liver circulation [39]. Due to ecological imbalance in vivo, intestinal barrier damage, and immune state change, bacterial products can reach the liver through the portal vein, where they are recognized by specific receptors, activate the immune system, lead to a pro-inflammatory reaction, and further aggravate the disease. On the other hand, intestinal flora, as a regulator of bile acid metabolism, also plays an important role in the process of fibrosis. It can metabolize primary bile acids into secondary bile acids, and further induce inflammation through the farnesoid X receptor (FXR), affect the immune response, and promote the process of liver fibrosis.

Thirdly, the liver–renal axis theory was used to explain the slowing of drug metabolism in the liver injury disease model [40]. Studies have shown that related factors produced in the process of chronic liver disease can lead to liver fibrosis, and cause kidney damage leading to chronic kidney disease. Chronic kidney disease (CKD) causes renal dysfunction, resulting in varying degrees of increase in the AUC of some drugs cleared by the kidney and a decrease in the drug clearance rate.

## 4. Materials and Methods

### 4.1. Plants, Chemicals, and Reagents

HPLC-grade formic acid was provided by DIKMA (Lake Forest, CA, USA). HPLC-grade methanol and acetonitrile were supplied by Thermo Fisher Scientific (Pittsburgh, PA, USA) and the water was purchased from Wahaha Group (Hangzhou, China).

Dried and ripped LJF were bought from Bozhou’s herbal market (Haozhou, China) and were stockpiled at the Heilongjiang University of Chinese Medicine. A representative specimen was deposited in the laboratory, and plant materials were authenticated by Prof. Lianjie Su from Heilongjiang University of Chinese Medicine, Harbin, China. The LJF sample was refluxed with ethanol:water (1:1, *v*/*v*) at a solid:liquid ratio of 1:10 for 2 h and then filtered. The residue was extracted again under the same conditions. The combined filtrates were concentrated under reduced pressure to remove the ethanol solvent. The aqueous solution was then freeze-dried under a vacuum. The dried powders were finally redissolved and dispersed in water for administration to rats.

The reference standards 3-CQA, 4-CQA, 5-CQA, 3,5-diCQA, 4,5-diCQA, Sweroside, Secoxyloganin, and daidzein (IS2) were purchased from Chengdu Must Biotechnology (Chengdu, China). The purity of each standard substance was higher than 98%. Furthermore, heparin sodium (Shanghai Pharmaceutical, Shanghai, China) was used for the mobile phase of chromatographic analysis and blood sample preparation. The chloramphenicol (IS1) was obtained from Sigma (St. Louis, MO, USA) with a purity of 99.99%.

### 4.2. Instrumentation

#### 4.2.1. Chromatographic Conditions

Eight compounds were separated on a Thermo Hypersil GOLD C18 column (100 mm × 2.1 mm, 1.9 µm) with the mobile phase consisting of (A) menthol and (B) 0.1% formic acid in water. The UPLC elution condition was optimized as follows: 0–6 min, 90% B–83% B, 6–7 min, 83% B–60% B, 7–12 min, 60% B–50% B, and 12–13 min, 50% B–90% B. The injection volume was set to 2 µL, and the column temperature was maintained at 45 °C.

#### 4.2.2. MS Conditions

MS analysis was adopted with a TSQ Quantis^TM^ Triple Quadrupole Mass Spectrometer (Thermo Fisher Scientific, USA) via selected reaction monitoring (SRM). A negative-ion scan was used to obtain the precursor ion and production spectra of phenolic acids, Secoxyloganin, and IS1, and a positive-ion scan was used to obtain the precursor ion and production spectra of Sweroside and IS2. Simultaneously, the data were acquired using Trace Finder TM software. The optimized parameters for 10 analytes (including IS1 and IS2) are shown in Table 1. Other optimized MS parameters were as follows: spray voltage 3500 V, the temperatures of the ion transfer tube and vaporizer were set at 325 and 350 °C, respectively, and the sheath gas and auxiliary gas pressures were 30 and 10 Arb, respectively.

### 4.3. Experiments on Animals

Specific acid-free male Sprague-Dawley rats with body weights of 250 ± 20 g were purchased from the Experimental Animal Center of Heilongjiang University of Chinese Medicine and fed in the SPF animal laboratory, under a standard room temperature of 25 ± 2 °C, relative humidity of 50 ± 15%, and a 12 h light/12 h dark cycle. The experiment followed the ethical norms of experimental animals of Heilongjiang University of Chinese Medicine (License number: SCXK (Hei) 2021-004). Twelve rats were randomly divided into normal and model groups of six rats each. After intraperitoneal injection of CCl_4_ (0.06 mL/kg) for 24 h, blood samples were collected into heparinized tubes via the orbital vein, centrifuged at 2000 r/min for 20 min, and the levels of AST and ALT in serum were determined by the microplate method.

### 4.4. Pharmacokinetic Studies

The pharmacokinetic properties and parameters of 3-CQA, 4-CQA, 5-CQA, 3,5-diCQA, 4,5-diCQA, PA, Secoxyloganin, and Sweroside in rats were studied after oral administration of the LJF extract. After the model was successfully induced, 0.83, 0.17, 0.25, 0.5, 1, 2, 4, 6, 8, 10, 12, 24, 36, and 48 h after administration of the ethanol extract of LJF (15.75 g/kg), 0.3 mL of blood was collected from the fundus venous plexus of rats and placed into a 1.5 mL centrifuge tube containing heparin sodium. The supernatant plasma was then transferred to a 1.5 mL centrifuge tube and stored at −20 °C for later use.

### 4.5. Correction Curves and Quality Control Samples Were Prepared

The 3-CQA, 4-CQA, 5-CQA, 3,5-diCQA, 4,5-diCQA, PA, Secoxyloganin, Sweroside, chloramphenicol, and daidzein were dissolved in methanol and added into the original standard solution at a concentration of 1 mg/mL. The original solution was continuously diluted with methanol:water (1:1, *v*/*v*). The internal standard solution of 500 ng/mL of chloramphenicol (IS1) and daidzein (IS2) and the gradient working solution of the analyte were obtained.

The standard curve samples were obtained by spiking the series standard solutions (100 μL) into blank rat plasma (100 μL) to yield the concentration ranges of 3-CQA, 4-CQA, 5-QCA, 3,5-diCQA, 4,5-diCQA, PA, Sweroside, and Secoxyloganin. The linearity ranges were: 25–13,500 ng/mL for 3-CQA, 7–3500 ng/mL for 4-CQA, 2.5–2100 ng/mL for 5-CQA, 30–5000 ng/mL for 3,5-diCQA, 7.5–2500 ng/mL for 4,5-diCQA, 3–300 ng/mL for PA, 15–6000 ng/mL for Sweroside, and 25–4500 ng/mL for Secoxyloganin.

### 4.6. Sample Preparation

Here, 100 μL aliquots of the plasma sample were thawed and pipetted into centrifuge tubes and 10 μL of chloramphenicol solution (500 ng/mL) and daidzein solution (500 ng/mL) were added, respectively, and 780 μL of methanol was added to precipitate for 5 min. Then, the supernatant was centrifuged for 5 min at 12,000 rp/min. The supernatant was transferred to a nitrogen blowing machine and dried at 40 °C. The residue was reconstituted in 100 μL of 50% methanol, and the sample was filtered through the membrane and then injected into the UPLC-MS/MS system.

### 4.7. Data Analysis

DAS 2.0 software was used to calculate the pharmacokinetic parameters of the non-atrioventricular model, including *C_max_*, *T_max_*, *AUC_0–t_*, *AUC_0–∞_*, *MRT_0–t_*, *MRT_0–∞_,* and *t_1_*_/*2*_. The statistical software GraphPad Prism 5.0 was used for graph plotting and statistical analysis of drug duration curves. The results are shown in Figure 3 and Table 1.

### 4.8. Method Validation

#### 4.8.1. Specificity

To assess obvious sources of interference at the retention times of interest in blank samples under the same operating conditions, the specificity of the values of the current method was investigated. The differences between the target analytes and interference were analyzed by comparing chromatograms from blank plasma and plasma spiked with target analytes and IS1 and IS2. The specificity was assessed by analyzing the chromatograms of blank plasma and plasma samples spiked with the eight components, IS1, and IS2, along with real plasma samples collected from LJF extract-treated groups.

#### 4.8.2. Linearity and Lower Limits of Quantification

Weighted least-square linear regression (1/x2) was used to plot the ratio of the peak area of the analyte to IS to the plasma concentration, and the correction curves of the eight analytes were constructed. The linear correlation coefficient should be greater than 0.99. Lower limits of quantification (LLOQ) were detected on the minimum analysis of six repeated plasma samples on the calibration curve. The signal-to-noise ratio of LLOQ should be greater than 5. The precision (RSD) and accuracy (RE) required by the LLOQ should be within 20% and 15%, respectively.

#### 4.8.3. Accuracy and Precision

Three different concentrations of QC samples were prepared. For each concentration, five samples were analyzed and measured for three consecutive days. The concentration of QC samples was calculated according to the standard curve of daily travel. The intra-day and intra-day precision values (expressed by RSD %) was not more than 15%, and accuracy (expressed as Re %) is required to be within ±15%.

#### 4.8.4. Recovery and Matrix Effect

Three QC samples of different concentrations were prepared for each compound. Comparing analytical results of analytes in extracted samples with corresponding extracts of blanks spiked with the analytes post-extraction. The matrix effect was measured by comparing peak areas of analytes in post-extracted samples with those acquired from pure solutions. The effect of the plasma matrix on the measured compounds and internal standard compounds was determined by comparing the peak areas of the standard solution and the standard solution with the same concentration after plasma samples’ treatment, as to ensure that the coefficient of variation was within 15%.

#### 4.8.5. Stability

For the measurement of stability of the eight analytes and IS in rat plasma, three QC samples of each concentration at low, medium, and high levels were prepared for analysis under different storage conditions. Stability in plasma was assessed by analyzing samples kept at room temperature for 8 h, samples stored at 80 °C for 30 days, and samples after 3 freeze–thaw cycles. The stability of the stock solutions was determined 24 h after storage at 4 °C. RE was used to express stability.

## 5. Conclusions

In this study, a rapid, sensitive, and selective method for simultaneous determination of two iridoid glycosides and six phenolic acids in rat plasma was established by UPLC-MS/MS. In addition, this method was successfully used for the first time in the pharmacokinetic study of eight analytes in normal rats and liver injury model rats after oral administration of LJF. There were significant differences in some pharmacokinetic parameters between the two groups, which might be related to the pathology of acute liver injury induced by CCl_4_ and the pharmacological effects of analytes. In addition, this study also provides a reference for further drug development and clinical application of LJF.

## Figures and Tables

**Table 1:** Quantitative ions and MS parameters of nine analytes and ISs.

Compounds	Retention Time (min)	Polarity	Precursor–Product Ion Transition	Collision Energy (Volts)
3-CQA	4.80	[M − H]^−^	*m*/*z* 353.038 → *m*/*z* 126.920; *m*/*z* 190.970	34.30; 16.75
4-CQA	5.11	[M − H]^−^	*m*/*z* 353.038 → *m*/*z* 173.042; *m*/*z* 178.970	15.65; 16.26
5-CQA	2.38	[M − H]^−^	*m*/*z* 353.038 → *m*/*z* 178.970; *m*/*z* 190.970	18.23; 18.87
3,5-diCQA	9.35	[M − H]^−^	*m*/*z* 515.000 → *m*/*z* 191.000; *m*/*z* 353.137	30.02; 15.31
4,5-diCQA	9.92	[M − H]^−^	*m*/*z* 515.138 → *m*/*z* 172.970; *m*/*z* 353.155	28.35; 17.92
PA	2.16	[M − H]^−^	*m*/*z* 152.862 → *m*/*z* 90.929; *m*/*z* 108.97	24.97; 14.36
Sweroside	8.39	[M + H]^+^	*m*/*z* 358.700 → *m*/*z* 126.971; *m*/*z* 179.042; *m*/*z* 197.000	24.52; 18.98; 10.23
Secoxyloganin	9.12	[M − H]^−^	*m*/*z* 404.075 → *m*/*z* 178.887; *m*/*z* 224.089; *m*/*z* 372.013	10.53; 15.50; 13.72
Chloramphenicol (IS1)	9.14	[M − H]^−^	*m*/*z* 321.000 → *m*/*z* 152.071; *m*/*z* 257.071	16.22; 10.23
Daidzein (IS2)	10.87	[M + H]^+^	*m*/*z* 254.825 → *m*/*z* 152.071; *m*/*z* 180.946 *m*/*z* 198.988	42.38; 31.87; 26.00

**Table 2:** The regression equations, linear range, and LLOQs for the eight compounds.

Analytes	Calibration Curves	r	Range (ng/mL)	LLOQ (ng/mL)
3-CQA	y = 0.0007x + 0.0019	0.996	25–13,500	25
4–CQA	y = 0.002x − 0.001	0.996	7–3500	7
5-CQA	y = 0.005x − 0.012	0.998	2.5–2100	2.5
3,5-diCQA	y = 0.002x − 0.004	0.994	30–5000	30
4,5-diCQA	y = 0.010x + 0.061	0.998	7.5–2500	7.5
PA	y = 0.002x + 0.001	0.995	3–300	3
Sweroside	y = 0.0004x − 0.007	0.990	15–6000	15
Secoxyloganin	y = 0.001x − 0.001	0.993	25–4500	25

**Table 3:** Intra- and inter-day precisions and accuracies for the determination of the eight compounds from the assay samples (mean ± SD, *n* = 6).

Analytes	Nominal Concentration (ng/mL)	Inter-Day		Intra-Day	
		Precision	Accuracy	Precision	Accuracy
		(RSD, %)	(Re, %)	(RSD, %)	(Re, %)
3-CQA	25	6.27	13.14	4.84	8.08
	65	2.89	−2.68	6.37	−1.57
	5000	7.88	−0.72	11.39	−7.01
	13,500	0.06	−5.95	0.09	−8.03
4-CQA	7	6.70	−9.41	7.48	−10.34
	27	9.37	9.94	12.41	13.77
	2000	5.05	−6.74	2.33	−6.65
	3500	3.19	−4.76	1.71	2.04
5-CQA	2.5	6.09	−11.42	4.02	−13.10
	9	10.62	0.94	9.61	−12.21
	800	2.52	−3.09	1.84	−4.70
	2100	12.52	10.06	3.40	4.24
3,5-diCQA	30	3.36	−3.21	4.10	−6.81
	50	8.54	0.02	3.99	−1.87
	3000	7.36	1.97	4.33	7.28
	5000	1.62	0.78	5.52	−4.93
4,5-diCQA	7.5	3.49	−8.61	3.61	−10.23
	15	15.63	−3.74	17.39	−9.18
	1300	3.59	−6.22	2.53	−3.15
	2500	3.95	−12.60	7.95	−13.20
PA	3	9.13	19.84	5.33	−4.32
	5	12.09	−14.34	11.01	−1.64
	180	9.03	−14.67	14.19	−12.14
	300	2.41	−7.23	5.55	−3.43
Sweroside	15	8.81	−9.13	11.03	−10.72
	40	7.34	−14.33	7.53	−12.61
	3600	8.78	−10.17	7.50	−10.34
	6000	6.92	−1.09	7.86	−0.49
Secoxyloganin	25	8.34	−15.87	6.87	−16.05
	40	16.10	−13.19	13.15	−9.19
	2600	1.17	−1.65	1.59	−6.61
	4500	2.59	−14.52	1.53	−12.58

**Table 4:** Matrix effects and extraction recoveries for the analytes and three internal standards in rat plasma (mean ± SD, *n* = 6).

Analytes	Spiked Concentration (ng/mL)	Recovery (%, *n* = 6)		Matrix Effect (%, *n* = 6)	
		Mean ± SD	RSD%	Mean ± SD	RSD%
3-CQA	65	91.28 ± 2.91	3.75	95.48 ± 4.17	9.38
	5000	86.34 ± 0.21	7.19	92.93 ± 3.85	4.14
	13,500	88.81 ± 6.45	7.26	96.36 ± 1.96	2.04
4-CQA	27	113.46 ± 11.09	9.78	93.29 ± 4.37	4.69
	2000	100.31 ± 5.07	5.05	90.68 ± 3.48	3.84
	3500	95.72 ± 6.94	7.25	92.96 ± 2.36	2.54
5-CQA	9	104.04 ± 10.92	1.05	85.74 ± 3.22	3.7
	800	102.16 ± 3.07	0.03	98.87 ± 3.09	3.13
	2100	108.59 ± 12.32	11.35	98.20 ± 4.79	4.88
3,5-diCQA	50	109.03 ± 12.74	11.68	90.48 ± 6.54	7.23
	3000	95.63 ± 12.76	13.35	92.22 ± 4.88	5.30
	5000	98.62 ± 5.61	5.69	87.25 ± 7.64	8.75
4,5-diCQA	15	97.48 ± 18.82	13.38	94.69 ± 4.27	4.51
	1300	101.57 ± 13.59	4.23	96.26 ± 2.56	2.66
	2500	85.56 ± 1.88	2.20	98.55 ± 2.22	2.25
PA	5	90.22 ± 7.99	8.85	90.88 ± 4.31	4.74
	180	89.21 ± 5.98	6.70	95.00 ± 5.02	5.29
	300	98.56 ± 4.85	4.92	92.31 ± 2.96	3.21
Sweroside	40	98.31 ± 11.33	11.52	90.30 ± 4.85	5.37
	3600	88.49 ± 6.69	7.56	100.61 ± 4.75	4.72
	4500	91.20 ± 7.35	8.06	96.39 ± 2.99	3.10
Secoxyloganin	40	96.68 ± 12.60	13.04	92.32 ± 5.63	6.10
	2600	99.37 ± 4.57	4.60	98.01 ± 2.88	2.94
	4500	98.05 ± 2.54	2.59	98.01 ± 12.31	12.56
Chloramphenicol (IS1)	500	94.95 ± 0.01	1.95	84.69 ± 0.34	4.11
Daidzein (IS2)	500	100.07 ± 0.10	9.49	99.05 ± 0.93	0.94

**Table 5:** The stability of the eight compounds in rat plasma under different storage conditions.

Analytes	Concentration (ng/mL)	25 °C for 4 h		Frozen for 30 Days		Three Freeze–Thaw Cycles		4 °C for 12 h	
		(RSD, %)	(Re, %)	(RSD, %)	(Re, %)	(RSD, %)	(Re, %)	(RSD, %)	(Re, %)
3-CQA	65	2.89	−2.68	6.31	−1.03	4.93	1.10	4.23	−2.19
	5000	7.88	12.49	2.09	17.46	3.36	8.73	7.77	12.49
	13,500	6.22	−5.95	3.06	−6.35	2.28	−2.45	10.15	−3.34
4-CQA	27	9.37	9.94	3.23	14.30	5.52	−14.36	12.30	−10.14
	2000	5.05	−6.74	11.75	−6.69	3.17	−2.52	5.72	−6.07
	3500	3.19	−4.76	2.59	−5.14	3.19	0.65	1.88	−3.52
5-CQA	9	10.62	0.94	7.74	−7.20	11.47	4.31	14.02	2.32
	800	2.52	−3.09	2.06	−6.61	1.95	−1.04	2.54	−4.57
	2100	12.53	10.07	5.54	−6.87	5.58	7.38	4.47	14.17
3,5-diCQA	50	8.54	0.02	6.75	−3.78	6.91	−5.47	13.71	−10.44
	3000	7.36	1.97	6.64	−7.91	7.10	13.19	12.37	7.24
	5000	1.63	0.78	2.40	−16.72	3.64	−19.50	10.19	5.89
4,5-diCQA	15	15.63	−3.74	15.19	−5.68	5.44	0.05	15.94	−11.51
	1300	3.59	−6.22	9.83	−10.26	3.15	−9.93	2.28	−12.55
	2500	3.95	−12.60	2.41	−14.53	11.35	−13.61	11.38	−12.28
PA	5	12.09	−14.34	11.37	−9.81	14.47	−8.02	7.39	−7.75
	180	9.03	−14.67	2.96	−5.75	2.00	−3.52	1.69	−6.94
	300	2.41	−7.22	7.52	−6.93	4.75	3.29	6.51	−9.11
Sweroside	40	7.34	−14.33	4.15	−12.88	14.83	−6.23	8.06	−4.82
	3600	8.77	−10.16	3.95	−15.16	5.21	−14.19	5.94	−10.82
	6000	6.92	−1.09	3.14	−3.77	3.93	−1.03	2.19	−1.19
Secoxyloganin	40	16.10	−13.19	12.18	−7.49	14.37	−9.66	13.89	−2.74
	2600	1.17	−1.65	3.43	−2.54	2.41	−3.5	2.30	−2.44
	4500	2.59	−14.52	1.37	−13.94	10.07	−11.58	9.04	−12.36

**Table 6:** Pharmacokinetic parameters of the eight compounds after oral administration of LJF extract in rats (mean ± standard deviation, *n* = 6).

Analytes	Group	*C_max_* (ng/mL)	*T_max_* (h)	*t_1_*_/*2*_ (h)	*AUC_0–t_* (ng/L)	*AUC_0–∞_* (ng/L)	*MRT_0–t_* (h)	*MRT_0–∞_* (h)
3-CQA	N	5113.7 ± 1222.21	0.2083 ± 0.04568	12.9 ± 0.765	12,104.7 ± 2285.95	13,124.31 ± 2298.26	13.92 ± 1.31	18.17 ± 1.94
	M	6286.14 ± 1262.72	0.19 ± 0.043	12.164 ± 3.12	16,356.04 ± 1719.08 **	17,442.89 ± 1551.76 **	13.45 ± 1.67	23.31 ± 11.25
5-CQA	N	265.59 ± 101.744	0.24 ± 0.03405	17.944 ± 9.969	1359.35 ± 249.57	1685.16 ± 730.52	17.96 ± 2.34	21.41 ± 2.86
	M	1115.21 ± 316.23 **	0.25 ± 0.13	18.86 ± 18.4	3568.54 ± 686.23 **	3970.61 ± 712.21 **	11.08 ± 2.42 **	20.92 ± 8.54
4-CQA	N	1108.35 ± 447.635	0.2083 ± 0.04568	15.17 ± 5.24	2174.6 ± 615.543	2335.247 ± 568.56	9.65 ± 1.72	14.73 ± 4.18
	M	2366.18 ± 425.38 **	0.14 ± 0.043 *	42.24 ± 31.66	5965.42 ± 1804.98 **	6809.52 ± 1139.85 **	10.14 ± 1.06	29.72 ± 24.81
3,5-diCQA	N	1228.21 ± 568.97	0.2222 ± 0.04307	21.186 ± 5.35	4452.3 ± 414.357	6081.8 ± 755.098	16.06 ± 1.42	22.87 ± 5.04
	M	3099.07 ± 1126.97 **	0.1944 ± 0.043	51.05 ± 47.28	6946.47 ± 898.12 **	11,219.19 ± 2773.706 **	13.71 ± 2.12 *	43.44 ± 35.17
4,5-diCQA	N	527.74 ± 333.416	0.18 ± 0.034	5.18 ± 0.69	1491.24 ± 634.12	1493.99 ± 633.17	12.12 ± 5.52	13.19 ± 5.2
	M	1579.03 ± 300.04 **	0.24 ± 0.14	50.79 ± 28.98 **	4017.55 ± 268.5 **	5898.58 ± 689.9 **	8.51 ± 0.72	41.21 ± 28.3 *
PA	N	22.56 ± 8.36	0.33 ± 0.13	24.79 ± 12.84	198.44 ± 36.97	292.41 ± 26.04	23.23 ± 0.86	44.148 ± 16.85
	M	23.00 ± 2.97	0.18 ± 0.034 *	25.46 ± 16.97	219.41 ± 59.07	546.58 ± 208.79 *	26.56 ± 2.13 **	49.79 ± 28.86
Sweroside	N	1928.45 ± 608.58	0.53 ± 0.38	9.78 ± 2.87	9685.09 ± 1410.29	10,003.33 ± 1540.57	12.29 ± 1.14	13.88 ± 1.86
	M	2364.23 ± 440	0.67 ± 0.26	11.846 ± 1.51	15,990.27 ± 903.4 **	16,726.12 ± 967.72 **	11.35 ± 0.48	13.69 ± 1.33
Secoxyloganin	N	1514.71 ± 331.078	0.46 ± 0.102	16.15 ± 19.09	6232.57 ± 1065.92	7613.15 ± 4120.64	11.89 ± 1.69	12.39 ± 1.04
	M	4154.96 ± 376.51 **	0.29 ± 0.102 *	23.56 ± 3.48	15,018.04 ± 2842.66 **	17,111.14 ± 3246.6 **	8.745 ± 0.58 **	18.2 ± 2.24 **

* Compared with the normal group (N), *p* < 0.05. ** Compared with the normal group (N), *p* < 0.01.

## Data Availability

All data generated or analyzed during this study are included in this article.

## References

[1] Liu T.L., Dong C.M., Gao Q.G., Zhang J.S., Qi D.M., Xu X.L., Li J.H. (2021). Brief discussion on textual research ideas of ancient books and textual collection of predecessors:A case study of Lonicera japonica. Chin. Tradit. Herb. Drugs.

[2] Wang Y.D., Yang J.B., Zhong Z., Ma S.C. (2014). Research progress on *Lonicerae japonicae flos*. Chin. J. Pharm. Anal..

[3] Liu S.T., Yang L., Wang S., Wang X.J., Zhang J.X., Hou A.J., Jiang H. (2020). Study on Chemical Components of Honeysuckle. Inf. Tradit. Chin. Med..

[4] Ehrman T.M., Barlow D.J., Hylands P.J. (2010). In silico search for multi-target anti-inflammatories in Chinese herbs and formulas. Bioorganic Med. Chem..

[5] Lou H.X., Lang W.J., Lv M.J. (1996). Separation and structure determination of water-soluble chemical compounds in *Lonicerae japonicae flos*. Chin. Tradit. Herb. Drugs.

[6] Zhang R., Wang T.T., Su P.W., Li M.M., Huang P., Li H.G. (2020). Study on Quality Standard of Organic Acid Effective Part of Honeysuckle. Asia-Pac. Tradit. Med..

[7] Huang L.J. (2006). Study on Synthesis of Bisantins Analogues from Lonicerae japonicae flos.

[8] Chen X.Y., Qin S.S., Li C., Wu Q.H., Jiang C., Yang J., Guo X.Y., Ou C.L. (2019). Differential Gene Expressions and Phytohormone Changes Altered Lonicera japonica Quality after Plant Introduction. World J. Tradit. Chin. Med..

[9] Zhang M., Ma X.Y., Xu H.L., Wu W.B., He X., Wang X.Y., Jiang M., Hou Y.Y., Bai G. (2020). A natural AKT inhibitor swertiamarin targets AKT-PH domain, inhibits downstream signaling, and alleviates inflammation. FEBS J..

[10] Rahman A., Kang S.C. (2009). In vitro control of food-borne and food spoilage bacteria by essential oil and ethanol extracts of Lonicera japonica Thunb. Food Chem..

[11] Li J., Ma X.B., Shen J., Zhang Z.F. (2020). Screening of active components from Chinese materia medica against SARS-CoV-2 based on literature mining and molecular docking. Chin. Tradit. Herb. Drugs.

[12] Liu J., Yan B.F., Zeng M.Y., Zeng Q.Q., Yang H.J., Zhang J.Z. (2020). Potential Mechanism of Couplet Medicines Baical Skullcap Root Honeysuckle for Coronavirus Disease 2019 Based on Network Pharmacology. World Chin. Med..

[13] Fu M.R., Qu Q.L., Dai H.F. (2015). Variation in antioxidant properties and metabolites during flower maturation of Flos Lonicerae Japonicae flowers. Eur. Food Res. Technol..

[14] Yin H.M., Lv X.Y. (2010). Study on preparation technology optimization and immune activity of Polysaccharide from Flos Lonicerae Japonicae flowers. China J. Chin. Mater. Med..

[15] Miao H., Zhang Y., Huang Z.L., Lu B., Ji L.L. (2019). Lonicera japonica Attenuates Carbon Tetrachloride-Induced Liver Fibrosis in Mice: Molecular Mechanisms of Action. Am. J. Chin. Med..

[16] Xu K.F., Deng L.H., Mao Z.H., Zhang X.R. (2021). Jinyinghua Oral Liquid in Combination with Interferon Spray for Treatment of Hand Foot and Mouth Disease in 40 Children with Pathogens Invading Lung-Spleen Pattern. J. Tradit. Chin. Med..

[17] Wang S., Yang L., Hou A.J., Liu S.T., Yang L., Jiang H., Kuang H.X. (2022). Screening hepatoprotective effective components of Lonicerae japonica Flos based on the spectrum-effect relationship and its mechanism exploring. Food Sci. Hum. Wellness.

[18] Qiao Y.R., Wang Z.H., Wang Y.F. (2021). Effect of pneumonia No.3 combined with honeysuckle granules on lung function, serum inflammatory factors and immune function in children with pneumonia. J. Guangxi Med. Univ..

[19] Zhang H.T., Chen J.L., Zhu X.Y., Zeng H.L., Li J., Sun X.B., Qi X.Y., Zeng J.C., Zeng Y.R. (2021). Effect and molecular mechanism of Honeysuckle-Rhizoma coptidis in the treatment of periprosthetic joint infection based on molecular docking and network pharmacology. Chin. J. Tissue Eng. Res..

[20] Fung J., Lai C.L., Yuen M.F. (2010). Hepatitis B virus DNA and hepatitis B surface antigen levels in chronic hepatitis B. Expert Rev. Anti-Infect. Ther..

[21] Asrani S.K., Devarbhavi H., Eaton J., Kamath P.S. (2019). Burden of liver diseases in the world. J. Hepatol..

[22] Zhang J.X., Li N., Xu Q.Y., Yang Y., Xie H.B., Shen T., Zhu Q.X. (2020). Kupffer cell depletion attenuates IL-6/STAT3 mediates hepatocyte apoptosis in immunological liver injury of trichloroethylene sensitized mice. Int. Immunopharmacol..

[23] Reuben A., Koch D.G., Lee W.M. (2010). Drug-induced acute liver failure: Results of a u.s. multicenter, prospective study. Hepatol. Off. J. Am..

[24] El-Kamary S.S., Shardell M.D., Abdel-Hamid M., Ismail S., El-Ateek M. (2009). A randomized controlled trial to assess the safety and efficacy of silymarin on symptoms, signs and biomarkers of acute hepatitis. J. Phytomed..

[25] Liang L., Bi Q., Dong J.C., Yang X.X., Yu J. (2018). Progress in the development of natural drugs with liver protection. Biot. Resour..

[26] Hu C.M., Jiang H., Liu H.F., Li R., Li J. (2007). Protective effect of Lonicera japonica total flavone (LTF)on immunological liver injury in mice. Pharmacol. Clin. Chin. Mater. Med..

[27] Chen H.L. (2011). Effects of Flos Lonicerae Japonicae on acute liver injury induced by carbon tetrachloride in mice. Chin. J. Gerontol..

[28] Wang D.S. (2011). Protective effect of extracts from Flos Lonicerae Japonicae on liver injury mice. Her. Med..

[29] Zhou Y.L., Zeng R., Pei Q., Liu S.K. (2016). Pharmacokinetics and bioavailability of chlorogenic acid extracted from Jinyinhua in rats. Chin. J. Hosp. Pharm..

[30] Kanhar S., Sahoo A.K. (2019). Meliorative effect of Homalium zeylanicum against carbon tetrachloride-induced oxidative stress and liver injury in rats. Biomed. Pharmacother..

[31] Yang C.C., Fang J.Y., Hong T.L., Wang T.C., Zhou Y.E., Lin T.C. (2013). Potential antioxidant properties and hepatoprotective effects of an aqueous extract formula derived from three Chinese medicinal herbs against CCl (4)-induced liver injury in rats. Int. Immunopharmacol..

[32] Lee K.J., Choi J.H., Jeong H.G. (2007). Hepatoprotective and antioxidant effects of the coffee diterpenes kahweol and cafestol on carbon tetrachloride-induced liver damage in mice. Food Chem. Toxicol..

[33] Zhu A.N., Li R., Liu S.H., Qiao Y.X., Shi S., Yuan S.X., Zhang J.P. (2014). Establishment of carbon tetrachloride-induced acute liver injury murine model. Chin. J. Liver Dis. (Electron. Version).

[34] Zhi Y., Song Y.N., Zhang M., Wang J.N., Zhao B. (2022). Pharmacological effects on carbon tetrachloride induced acute liver injury in mice. Chin. Arch. Tradit. Chin. Med..

[35] Xie Y., Hao H., An K., Yan L., Xie T., Sun S., Dai C., Zheng X., Lin X., Li J. (2010). Integral pharmacokinetics of multiple lignan components in normal, CCl4-induced hepatic injury and hepatoprotective agents pretreated rats and correlations with hepatic injury biomarkers. J. Ethnopharmacol..

[36] Li P., Lu Q., Jiang W., Xue P., Hao K. (2017). Pharmacokinetics and pharmacodynamics of rhubarb anthraquinones extract in normal and disease rats. Biomed. Pharmacother..

[37] Du G.F., Dong J.K., Zhang T., Yang X.R., Lu S.S., Qu J.H., Lu Y.Y., Hong Z.X. (2018). Correlation between Fyn and bile acid metabolism in liver fibrosis mice. Infect. Dis. Inf..

[38] Li L., Wang Y.L., Qing H.Y., Qu Y.Y., Yang T.S., Wang Z.Y., Xie J.R. (2021). Research progress on berberine in treatment of nonalcoholic disease by regulation gut-liver axis. Chin. Tradit. Herb. Drugs.

[39] Li Y.T., Wang L., Chen Y., Chen Y.B., Wang H.Y., Wu Z.W., Li L.J. (2010). Effects of Gut Microflora on Hepatic Damage After Acute Liver Injury in Rats. J. Trauma.

[40] Wang M.L., Ding Y.F., Yin X., Shao J.Z., Zhuang Z.R., Zhang T., Su P.L., Peng Y.R. (2021). Progress in understanding hepatic fibrosis and renal fibrosis based on the gut-liver-kidney axis. Acta Pharm. Sin..

